# Elongated Styloid Process and Cervical Spondylosis

**DOI:** 10.4137/ccrep.s792

**Published:** 2008-05-27

**Authors:** Zeliha Unlu, Sebnem Orguc, Gorkem Eskiizmir, Asim Aslan, Petek Bayindir

**Affiliations:** 1 Celal Bayar University School of Medicine Department of Physical Medicine and Rehabilitation.; 2 Celal Bayar University School of Medicine Department of Radiology.; 3 Celal Bayar University School of Medicine Department of Otorhinolaryngology.

**Keywords:** cervical, osteophyte, dysphagia, eagle’s syndrome

## Abstract

**Background:**

Dysphagia, is a significant sign of many different lesions in upper digestive system especially in proximal esophagus. Tumors, gastroesophageal reflux, achalasia and extrinsic compressions are the most common causes that may lead to dysphagia in geriatric population. Cervical osteophyte induced dysphagia, is one of the uncommon reasons of dysphagia, therefore other causes of dysphagia must be excluded to establish the exact diagnosis. Eagle syndrome is one of the considerable reason which may lead to misdiagnosis in patients with cervical osteophytes. In this case report, we represent four patients who had dysphagia due to anteriorly located cervical osteophytes and evaluate the patients with special reference to Eagle syndrome.

**Material and methods:**

After a detailed anamnesis and ENT examination, cervical plain radiographs in four projections and Towne radiographs were obtained for every patient. After that, magnetic resonance imaging (MRI) of cervical spine and barium swallowing studies were performed to evaluate the presence of esophageal compression.

**Results:**

Eagle syndrome was excluded due to absence of other symptoms and physical signs, eventhough unilateral or bilateral elongation of styloid processes was found in all of the patients.

**Conclusion:**

Cervical osteophytes induced dysphagia is a rare clinical entity, diagnosis should be done by a careful examination, intensive radiologic evaluation. Moreover, all the other causes like Eagle syndrome should be excluded during the diagnosis of cervical osteophyte induced dysphagia.

## Background

Cervical osteophytes have been reported to be one of the rare causes, despite of its high prevalance (20%–30%) in elderly population (1). The exact reason of cervical osteophyte induced dysphagia is not clarified yet, but direct compression or inflammation due to cervical osteophytes are claimed as the main reasons.

In our knowledge, Eagle syndrome, which is caused due to elongation of the styloid process or ossification of the stylohyoid ligament, and lead to recurrent throat pain, ipsilateral otalgia, foreign body sensation, dysphagia, facial pain, carotodynia, intermittent frontal or temporal headaches, and dizzy or blackout spells; should also be evaluated as a reason for external compression in patients with cervical osteophyte induced dysphagia. Nevertheless, whenever we surveyed the English literature, we have observed the lack of styloid process evaluation in patients with dysphagia induced by diffuse idiopathic skeletal hyperostosis (DISH) or cervical spondylosis. Therefore, we represented and discussed the evaluation of four patients with cervical osteophytes induced dysphagia with special reference to Eagle syndrome.

## Case Descriptions

Four patients with dysphagia due to cervical spondylosis were included in this report. All of our patients suffered from moderate to severe cervical pain and stiffness. There was no evidence of cervical lymphadenopathy, and palpation of the thyroid gland was normal in all cases. Neurologic examination of the patients was normal. Plain radiographs of the cervical and thoracic spine showed a massive ankylosing ossification of the anterior longitudinal ligament suggestive of DISH in case 3 and 4, and cervical spondylosis in case 1 and 2. The styloid process was considered to be elongated (abnormal) when the entire osseous length of the bony process and/or the mineralized portion of the stylohyoid ligament exceeded 25 mm on the Towne radiographs (3). There is no role of Magnetic resonance imaging (MRI) in showing the elongated styloid processes and thus establishing the diagnosis of Eagle syndrome. However MRI of the cervical spine (T1-w and T2-w sequences in sagittal and axial planes) which is an excellent tool in demonstrating the extuberant osteophytes and barium swallow studies searching the presence of compression of the esophageal lumen were performed. In ENT examinations the styloid processes were evaluated as normal with no physical examinations findings compatible with Eagle’s syndrome. Endoscopic evaluations of the esophagus were also performed and were completely normal in all cases. All blood work was within normal limits, including complete cell count and thyroid function testing, except hyperglycemia in the first (162 mg/dL) and third (372 mg/dL) patients.

### Patient 1

A 49-year-old man had episodes of “choking” with solids and a sensation of blockage which only revealed by liquid intake for the last 3 years. He had no history of gastroesophageal reflux symptoms and no remarkable weight loss. The patient denied any other symptoms, including odynophagia, dysphonia or dyspnea.

The lengths of styloid processes were elongated (right: 44 mm, left: 44 mm) in the Towne radiographs (Fig. 1a). Lateral cervical spine radiograms revealed exuberant osteophytes that projected anteriorly at C5–7 levels. A barium swallow showed a bridging osteophyte between C6 and C7 vertebrae indenting the esophagus posteriorly and displacing it anteriorly (Fig. 1b). Cervical MRI demonstrated focal cervical spurs at the ventral portion of the C5–7 vertebral bodies, protruding ventrally and compressing mildly to the esophagus (Fig. 1c). Videofluoroscopic examination of swallowing function showed mild congestion of the contrast medium at the portion of the epiglottic vallecula, without a sign of aspiration.

### Patient 2

A 56-year-old man suffered from progressive difficulty in swallowing of solid meals predominantly and a feeling of suffocation for two years, which especially increased in the last six months. He also has some complaints related to gastroesophageal reflux which occasionally became symptomatic, therefore lansoprasol 30 mg/day was started for symptoms of gastroesophageal reflux. He described difficulty in breathing and snoring while lying in supine position. All these symptoms accompanied dysphagia for the last 2–3 years. He had a history of an intubation difficulty during anesthesia for lumbar disc herniation surgery one year ago. There were no history of hoarseness, odynophagia and no remarkable weight loss.

The length of styloid process was measured in the upper limits at the right side (25 mm) while it was elongated at the left side (34 mm) (Fig. 2a). A barium swallow test (Fig. 2b) demonstrated osteophytic spurs in the anterior aspect of C4–5 vertebrae, which were so close to the inlet of esophagus, and obstructing the esophagus passage by external compression. Cervical MRI demonstrated giant cervical spurs at the ventral portion of the C4–C7 vertebral bodies, protruding ventrally and compressing to the esophagus (Fig. 2c).

### Patient 3

A 62-year-old woman presented with a six-month history of an increasing dysphagia and weight loss of approximately 7 kg, with associated chest pain during swallowing and odynophagia. The patient also complained of symptoms consistent with aspiration. She described difficulty in breathing and snoring while lying in supine position.

Similar to the second case, the length of styloid process was in the upper limits at the right side (25 mm) while it was found elongated at the left side (35 mm) (Fig. 3a). Barium swallow test revealed indentations along the pharynx at multiple levels, which were more prominent at C3–4 and C4–5 (Fig. 3b). Cervical MRI showed excessive anterior osteophyte formations in multiple levels and compression of esophagus due to ossificated anterior longitudinal ligament (Fig. 3c).

### Patient 4

A 68-year-old woman presented with a history of an increasing dysphagia for 5 years, especially with solid foods and aspiration. She had no weight loss.

The length of styloid process were elongated on both sides (right: 29 mm, left: 30 mm) (Fig. 4a). Barium swallow test revealed indentations along the esophagus, which were more prominent at C5–6 and C6–7 (Fig. 4b). Cervical MRI showed anterior osteophyte formations at the same levels which compressed the esophagus (Fig. 4c).

## Discussion

Dysphagia is an associated sign for both cervical osteophyte induced dysphagia and Eagle syndrome. However, this situation is not decently evaluated in the literature. Therefore, during the diagnosis of cervical osteophyte induced dysphagia Eagle syndrome should be excluded. The diagnosis of Eagle syndrome can usually be made on physical examination by digital palpation of the styloid process in the tonsillar fossa which exacerbates the pain (2). Our cases did not describe other more typical symptoms for this syndrome except the dysphagia. They did not report any pain during the palpation of tonsillar fossa on ENT examination; so we have excluded Eagle syndrome.

It was stated that elongated styloid processes occur more frequently in older patients (3). This is explained by degeneration of the ligamentous apparatus linked to a general tendency toward the deposition of calcium salts (4). Osteophyte is also a common disorder in geriatric population; and ossification of the ligamentous or bony structures is the main reason in both of the diseases. Guo et al. (5) stated that various types of ossification as well as enlargement of the styloid process and the stylohyoid ligament correlate significantly with ligamentous ossification or osteophytes of the cervical spine, and such correlation could explain the presence of clinical findings compatible with Eagle’s syndrome among some patients with DISH involving the cervical spine. Our cases emphasize a relationship between osteophytes and elongation of the styloid process. Elongated styloid process may lead to Eagle syndrome and this syndrome could be another cause of dysphagia. Therefore, it should be excluded during the diagnosis of the patients with cervical osteophyte induced dysphagia.

## Conclusion

In conclusion the presence of Eagle’s syndrome and combined role in the etiology of dysphagia should be considered during the process of diagnosis in osteophyte related dysphagia.

## Figures and Tables

**Figure 1a:**
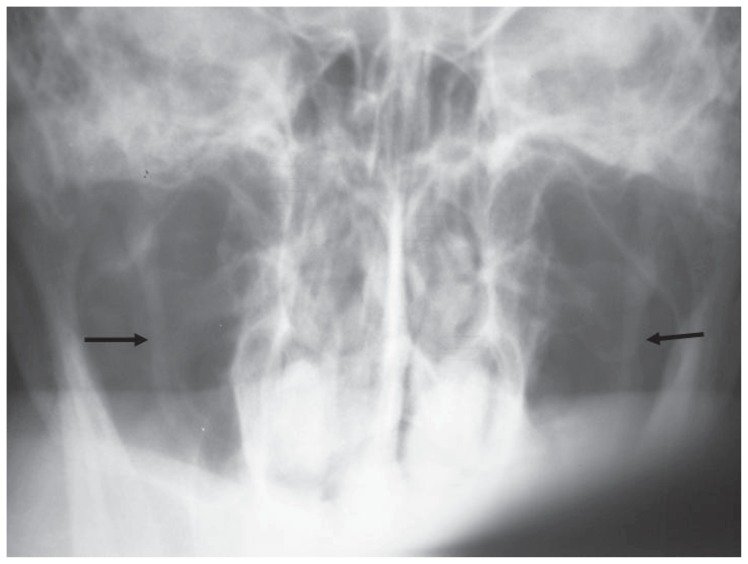
Towne radiograph shows bilateral elongated styloid processes (right: 44 mm, left: 44 mm).

**Figure 1b:**
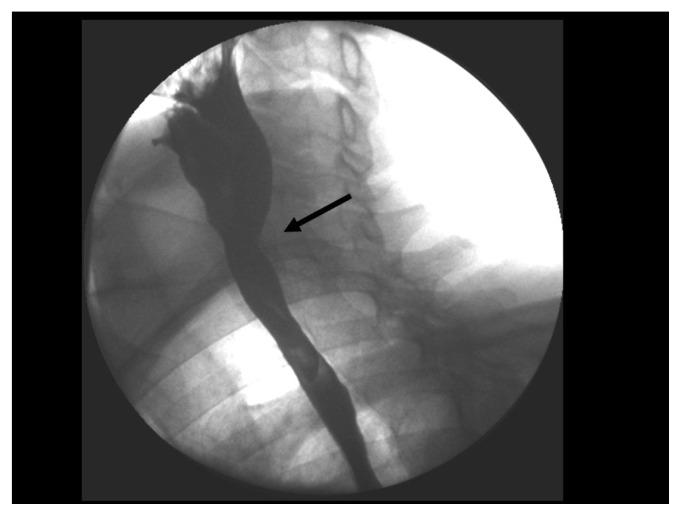
Videoflouroscophic examination confirms posterior extrensic compression of the barium column by osteophytes at the level of C6–7 and proximal dilatation of the esophagus.

**Figure 1c:**
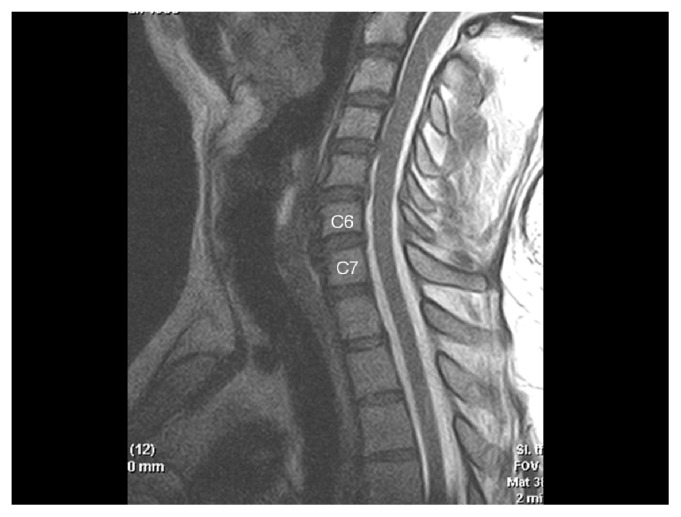
Sagittal T2-w MRI showing compression on the esophagus caused by anterior osteophytes.

**Figure 2a:**
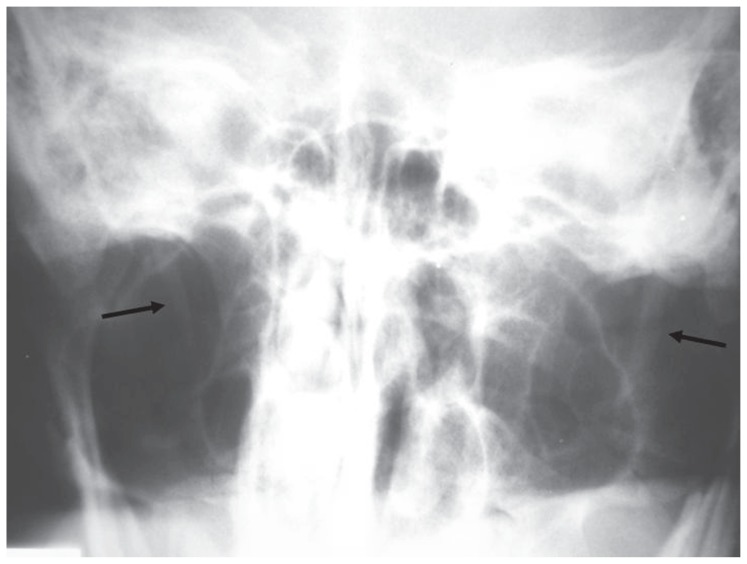
The length of styloid process was measured in the upper limits at the right side (25 mm) while it was elongated at the left side (34 mm).

**Figure 2b:**
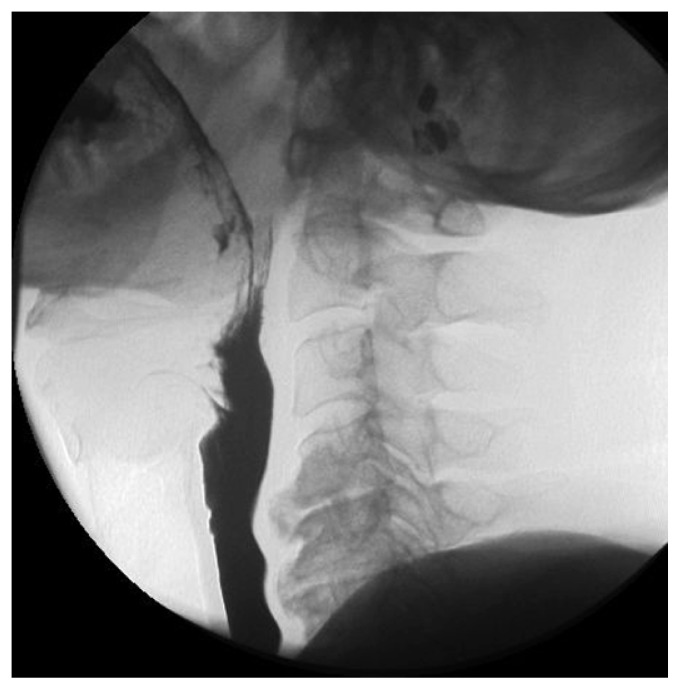
Videoflouroscophic examination of the esophagus showing compression by osteophytes at C4–5 and C5–6 levels.

**Figure 2c:**
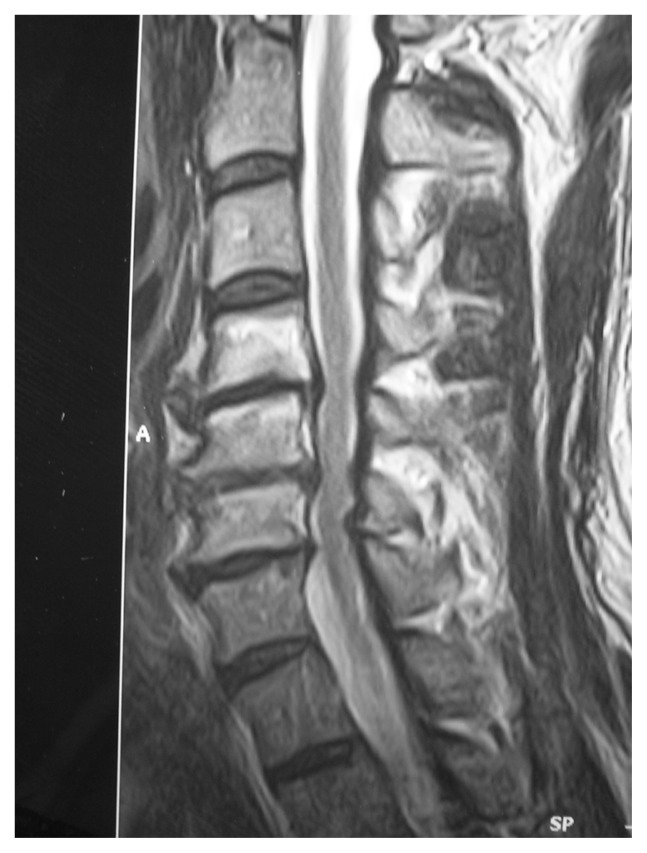
Sagittal T2-w MRI demonstrating degenerative disc protrusions and anterior osteophytes compressing on the esophagus at C4–7 levels.

**Figure 3a:**
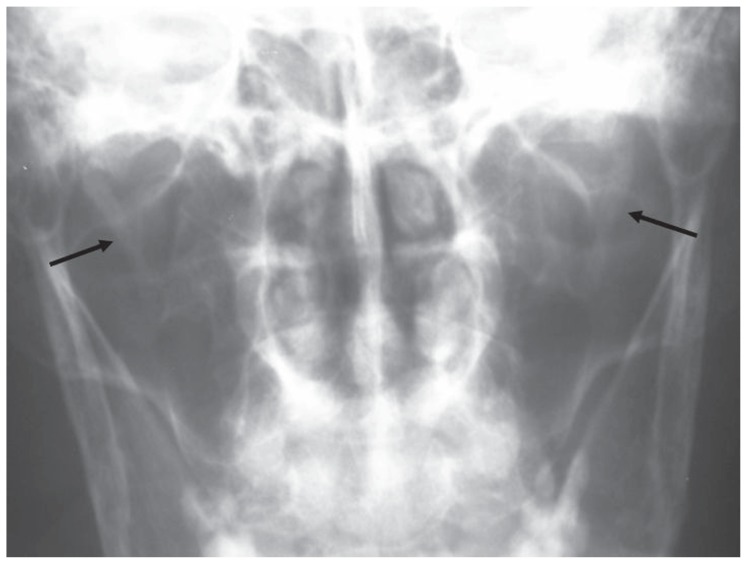
The styloid process was in the upper limits at the right side (25 mm) and elongated at the left side (35 mm).

**Figure 3b:**
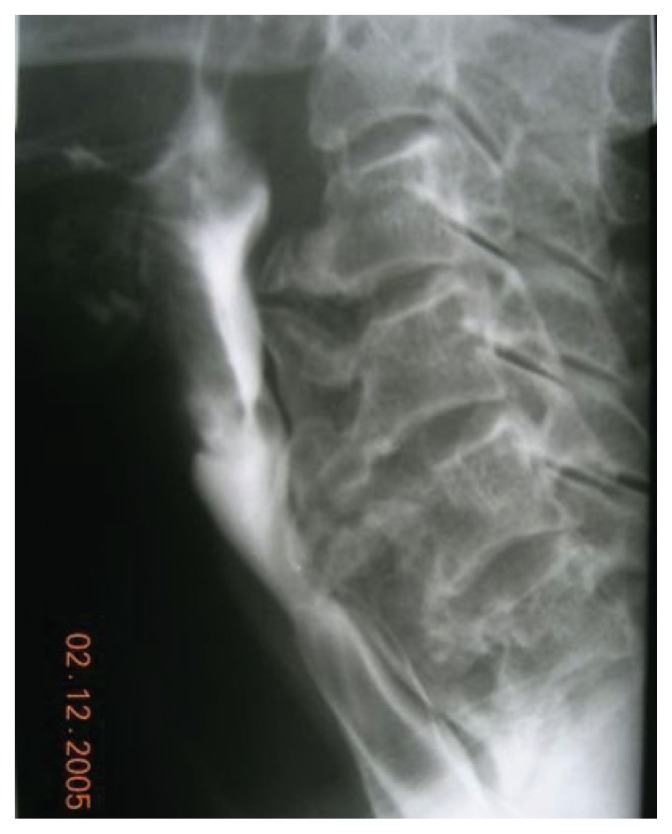
Barium swallow showing indentations on the esophagial lumen caused by anterior osteophyte formations and ossificated anterior longitudinal ligament more prominent at C3–4 and C4–5 levels.

**Figure 3c:**
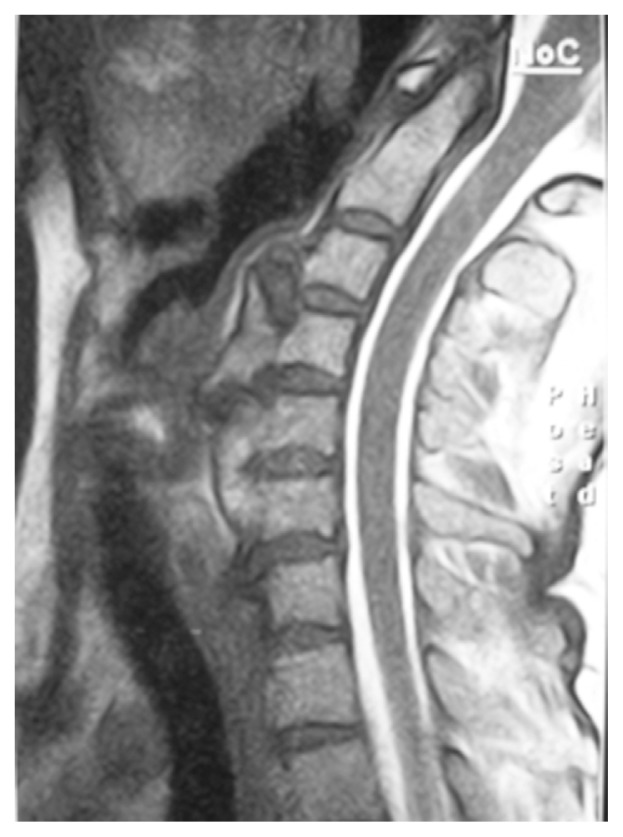
Sagittal T2-w MRI demonstrating excessive anterior osteophyte formations and ossificated anterior longitudinal ligament compressing on the esophagus at multiple levels.

**Figure 4a:**
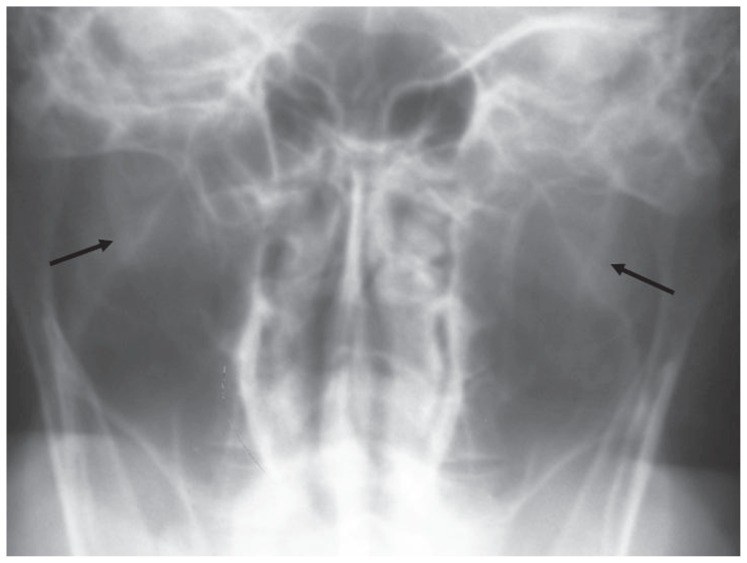
Towne radiograph shows bilateral elongated styloid processes (right: 29 mm, left: 30 mm).

**Figure 4b:**
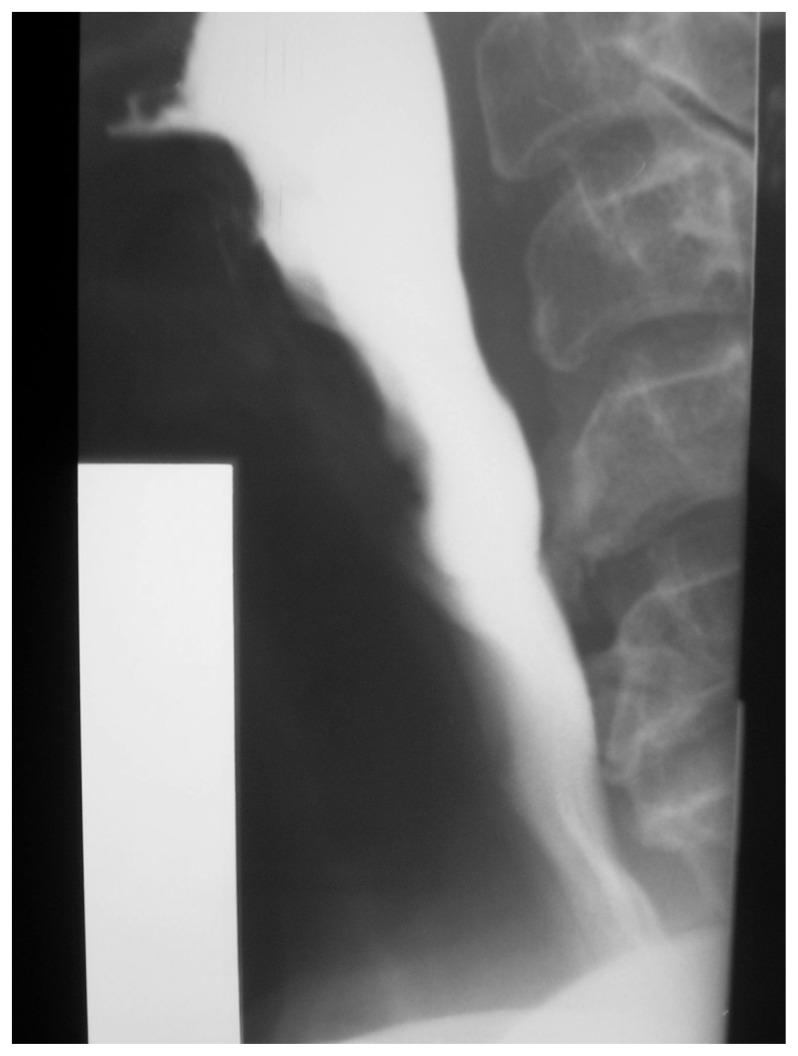
Barium swallow showing indentations on the esophagial lumen caused by anterior osteophyte formations more prominent at C5–6 and C6–7 levels.

**Figure 4c:**
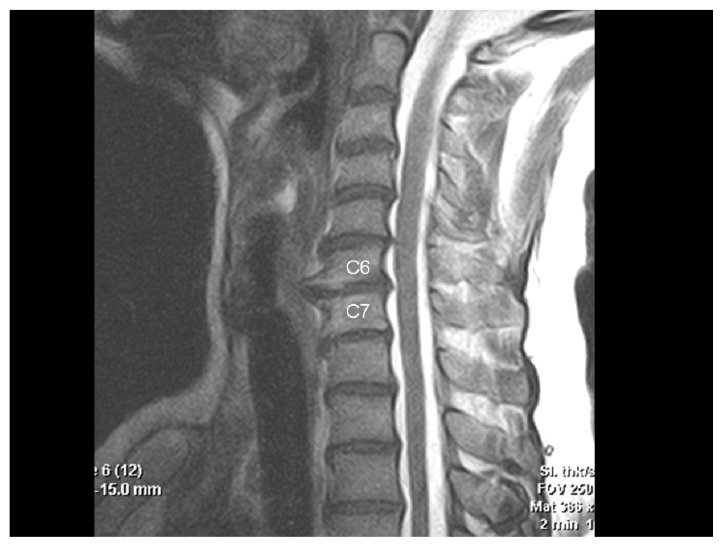
Sagittal T2-w MRI demonstrates compression of the esophagus by anterior osteophytes.
